# Phylogenetic Analysis of the Synnema-Producing Genus *Synnemapestaloides*

**DOI:** 10.3390/jof2040028

**Published:** 2016-11-07

**Authors:** Kyoko Watanabe, Mao Sekiguchi, Toyozo Sato, Tom Hsiang, Shigeru Kaneko, Kazuaki Tanaka, Masaru Kanda, Naoko Fujita, Shunsuke Nozawa

**Affiliations:** 1 Faculty of Agriculture, Tamagawa University, 6-1-1 Tamagawa-gakuen, Machida, Tokyo 194-8610, Japan; 2 Graduate School of Agriculture, Tamagawa University, 6-1-1 Tamagawa-gakuen, Machida, Tokyo 194-8610, Japan; ma000.sanns@gmail.com (M.S.); nzwsh1ba@agrs.tamagawa.ac.jp (S.N.); 3 Mycology & Metabolic Diversity Research Center, Tamagawa University, 6-1-1 Tamagawa-gakuen, Machida, Tokyo 194-8610, Japan; 4 Genetic Resources Center, National Agriculture and Food Research Organization, 2-1-2 Kannondai, Tsukuba, Ibaraki 305-8602, Japan; s1043@gene.affrc.go.jp (T.S.); skaneko@a.email.ne.jp (S.K.); 5 Environmental Sciences, University of Guelph, Guelph, ON N1G 2W1, Canada; thsiang@uoguelph.ca; 6 Faculty of Agriculture and Life Sciences, Hirosaki University, 3 Bunkyo-cho, Hirosaki, Aomori 036-8561, Japan; k-tanaka@hirosaki-u.ac.jp; 7 Green Doctors, 1-10-13-201 Sagamihara, Chuoku, Sagamihara, Kanagawa 252-0231, Japan; kanda-m@bp.iij4u.or.jp; 8 Laboratory of Plant Pathology, Graduate School of Agriculture, Tokyo University of Agriculture and Technology, 3-5-8 Saiwaicho, Fuchu, Tokyo 183-8509, Japan; afujita@cc.tuat.ac.jp

**Keywords:** Amphisphaeriales, conidiomatal development, Sporocadaceae, *Synnemapestaloides*

## Abstract

*Synnemapestaloides rhododendri*, the type species of the genus *Synnemapestaloides*, is a pathogen of *Rhododendron brachycarpum*. This fungus produces six-celled conidia with appendages at both end cells, and are generated by annellidic conidiogenous cells on the synnema. These conidial structures are similar to those of the genus *Pestalotia*. The monotypic genus *Synnemapestaloides* is currently classified in the family Amphisphaeriaceae solely based on conidial morphology. Here we demonstrate that *Synnemapestaloides* represents a distinct genus in the family Sporocadaceae (Amphisphaeriales) based on differences in the nucleotide sequences of the partial large subunit rDNA gene, the rDNA internal transcribed spacer, and the partial *β-tubulin.* The genus most closely related to *Synnemapestaloides* is *Seimatosporium* and the species most similar to *Synnemapestaloides rhododendri* is *Seim. foliicola* which produces short synnema-like conidiomata (sporodochia). These results demonstrate that *Seim. foliicola* should be transferred to *Synnemapestaloides*, and also demonstrate that Sporocadaceae can have synnematal in addition to pycnidial and acervular conidiomata.

## 1. Introduction

Pestalotioid fungi belong to genera such as *Pestalotia*, *Pestalotiopsis*, *Monochaetia*, and *Seimatosporium*, among others. These genera are characterized by septate conidia with appendages that are apical, basal, or both, which are produced from annellidic conidiogenous cells lining the upper and inner layer of acervuli and pycnidia [1,2]. Because of the presence of acervuli or pycnidia, these fungi are all considered coelomycetes. Their formal classification as genera in Sporocadaceae is supported by similarities among sequences of their nuclear ribosomal RNAs [3,4,5,6,7,8].

*Synnemapestaloides rhododendri* T. Handa & Y. Harada was isolated from twig blight of *Rhododendron brachycarpum* D. Don ex G. Don. It was described as a new genus and species by Handa et al. [9], and has only been found in Japan. The conidia of this fungus are six-celled, including four pigmented median cells, and have branched appendages at the distal and basal cells. Thus, conidial morphology is the same as that of the genus *Pestalotia* [10]. The presence of percurrently extending conidiogenous cells is an ontogenetic pattern common among pestalotioid fungi. The major phenotypic difference between *Synnemapestaloides* and *Pestalotia* is their conidiomatal morphology, *Synnemapstalotiopsis* produces synnemata and *Pestalotia* produces acervuli.

Hyde et al. [11] published a list of anamorphic names and, without further explanation, placed *Synnemapestaloides* in the family Amphisphaeriaceae together with pestalotioid fungi. However, the name *Synnemapestaloides* has not been used in research on pestalotioid fungi since then, and it has not been included in subsequent phylogenetic analyses. Jaklitsch et al. [8] transferred pestalotioid fungi to the Sporocadaceae from Amphisphaeriaceae except for *Synnemapestaloides*. *Synnemapestaloides* may belong in the Sporocadaceae; however, more information and evidence is needed to properly place this genus—*Synnemapestaloides* may belong to the Amphisphaeriaceae, Sporocadaceae, or another family, but clear evidence is still lacking. There are no synnematal fungi known in the Amphisphaeriaceae nor in the Sporocadaceae.

To define the phylogeny of *Synnemapestaloides,* we analyzed the nucleotide sequences encoding the partial large subunit rRNA gene (LSU), the internal transcribed spacer (ITS), and a portion of *β-tubulin*, and additionally conducted morphological analyses. The relationship between *Synnemapestaloides* and other pestalotioid fungi is discussed here.

## 2. Materials and Methods

### 2.1. Strains

The morphological and phylogenetic analyses included eight strains of *Syn. rhododendri* isolated from *Rhododendron brachycarpum* (including the ex-type strain) and three strains of *Seimatosporium* (Table 1). These strains were deposited in the herbarium of Hirosaki University (HHUF) as well as the culture collections of Tamagawa University (TAMA), Genebank Project NARO, Japan (MAFF). These strains were grown on potato dextrose agar (PDA) (Eiken, Tochigi, Japan) at 25 °C.

### 2.2. DNA Extraction and Polymerase Chain-Reaction (PCR)

DNA from each fungal strain was extracted from one-week-old PDA cultures using a Qiagen DNA Mini Kit (Qiagen, Tokyo, Japan) following the manufacturer’s protocol. The genes amplified with these PCR primers: (1) ~650 bp of the LSU (D1–D2 region) with primers NL1 and NL4 [12]; (2) ca 540 bp of the ITS with primers ITS4 and ITS5 [13]; and (3) ~490 bp of β-tubulin with primers BT2a and BT2b [14]. PCR products were purified using ExoSAP-IT reagent (GE Healthcare Japan, Tokyo, Japan) and sequenced using an ABI 310 DNA sequencer (ABI, Tokyo, Japan). These sequences were deposited in the DNA Data Bank of Japan (accession numbers shown in Table 1).

### 2.3. Molecular Phylogeny

To compare amplicon sequences, data obtained from online sources and taxa (including *Discostroma,* which is the sexual morph of *Seimatosporium*) were mainly chosen from previous research on phylogeny of Pestalotioid fungi such as Jaklitsch et al. [8] and Tanaka et al. [6] (Table 1). Sequences were aligned using ClustalW [15] implemented in BioEdit [16], and manually optimized. The alignments were subjected to phylogenetic analysis using MEGA software version 7 [17]. All positions containing gaps and missing data were not considered for analyses. The strength of the internal branches from the tree was tested using bootstrap analysis [18] with 1000 replications. Trees were viewed using TreeView [19], and evolutionary history was inferred using the maximum parsimony (MP) method [20]. Consistency, retention, homoplasy, and composition indices were calculated for parsimony-informative sites.

MP trees were generated using the Subtree-Pruning-Regrafting algorithm [20] and search level 5, which generates initial trees by randomly adding sequences (10 replicates). Molecular analyses using the maximum likelihood (ML) method were performed The *T92+G+I* nucleotide substitution model based on the Tamura three-parameter model [21] for ITS plus LSU phylogenetic analysis and the Kimura two-parameter method [22] for ITS plus *β-tubulin* analysis. Initial trees for the heuristic search were automatically generated by applying the neighbor-joining (NJ) and BioNJ algorithms to a matrix of pairwise distances estimated using the maximum composite likelihood approach and then selecting the topology with a higher log-likelihood value. Evolutionary history was inferred using the NJ method [23]. The tree was drawn to scale with branch-length units equivalent to those of the evolutionary distances used to infer phylogeny. Evolutionary distances were computed using the Kimura two-parameter method [22] and are expressed as the number of base substitutions per site.

To generate phylogenies based on ITS plus LSU sequences, *Lepteutypa fuckelii*, *Lepteutypa sambuci*, and *Phlogicylindrium uniforme* which are members of Amphisphaeriaceae (outgroup) and Phlogicylindriaceae and were chosen since they are phylogenetically close to Sporocadaceae [8]. For phylogenies assessed using ITS and *β-tubulin* sequences that included a more limited selection of taxa, *Discosia artocreas* (Tode) Fr. served as the outgroup. The data of trees are deposited in TreeBase (S19936 for ITS + LSU, S19927 for ITS + *β-tubulin*).

### 2.4. Morphological Observations

Eight strains of *Syn. rhododendri* were cultured for 30 d at room temperature on PDA containing the leaves of *H. macrophylla* [24]. Light microscopy (B51, Olympus Tokyo, Japan) was used to assess characteristics of conidia.

A stereomicroscope (WILD10; Leica Geosystems, Tokyo, Japan) was used to observe the development of conidiomata on a simple agar medium amended with a leaf piece of *H. macrophylla*. Specimens were fixed with a modified method [24] before analysis by scanning electron microscopy. Conidiomata were soaked in 0.2% osmium tetroxide for 1 h at 4 °C, washed with 0.2 M phosphate buffer (pH 7.2), and resoaked in 0.2% glutaraldehyde overnight at 4 °C. These specimens were washed again in 0.2 M phosphate buffer and dehydrated using an ethanol series. They were then critically dried using an Eiko DX-1 apparatus (Eiko, Tokyo, Japan), coated with gold using a JEOL JFC 1100 sputtering system (JEOL, Tokyo, Japan), and examined using a scanning electron microscope (JEOL 5200, JEOL, Tokyo, Japan) at 20 kV.

## 3. Results

### 3.1. Phylogenetic Analysis of the ITS and LSU D1–D2 Region

**Figure 1:**
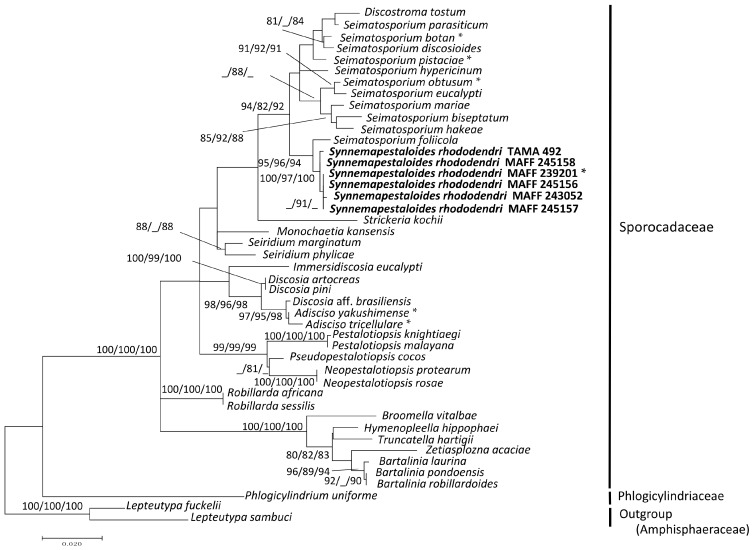
Maximum likelihood (ML) tree with the highest log-likelihood (−4141.69) determined by analysis of the combined ITS and LSU (D1–D2) sequence matrix. Numbers (ML/MP/NJ) and hyphens on the branches indicate the bootstrap values (%) for each node, calculated from 1000 replicates and only values >80% are shown. MP: maximum parsimony, NJ: neighbor-joining. *: Ex-holotype cultures. Strains in Bold were investigated in this study.

Thirty-six strains of pestalotioid fungi, including one preserved and five fresh strains of *Syn. rhododendri*, were examined (accession numbers shown in Table 1). The sequence matrix used for phylogenetic analyses contained 575 bp from ITS and 542 bp from LSU. Each analysis included at least 949 nucleotide positions (416 bp of ITS and 533 bp of LSU), and one of the three most parsimonious trees (length = 509, these are similar topology) of the MP tree analysis using the Subtree-Pruning-Regrafting is shown in Figure S1A. The consistency, retention, and composite indices were 0.454, 0.783, and 0.35th the highest log-likelihood (−4141.69), respectively. The optimal tree generated using the NJ method had a branch-length sum = 0.495 (Figure S1B). Only the ML tree (Figure 1) is shown here, since the MP and NJ methods generated similar topologies.

The six strains of *Syn. rhododendri* with synnemata were placed with other pestalotioid fungi. In this family, *Synnemapestaloides* and *Seimatosporium* were in the same clade with high bootstrap support (ML/MP/NJ: 94/82/92), and *Syn. rhododendri* was included in a subclade with high bootstrap support as well (ML/MP/NJ: 100/97/100). The species most closely placed with *Synnemapestaloides* was *Seim. foliicola* with high support (ML/MP/NJ: 95/96/94).

### 3.2. Phylogenetic Analysis of ITS and β-Tubulin

**Figure 2:**
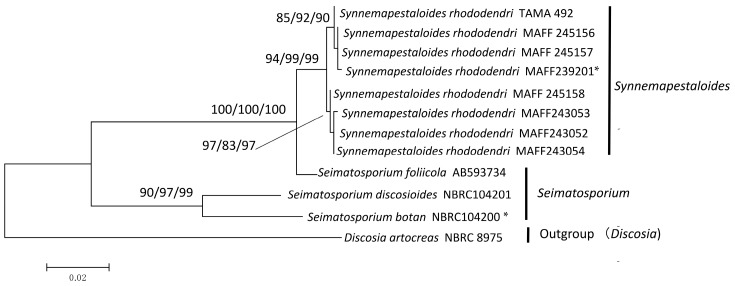
Maximum likelihood (ML) tree with the highest log-likelihood (−2131.68) determined by analysis of the combined ITS and *β-tubulin* sequence matrix. The numbers (ML/MP/NJ) on the branches indicate bootstrap values (%) for each node, calculated from 1000 replicates. MP: maximum parsimony, NJ: neighbor-joining. *: Ex-holotype cultures.

The sequences of eight *Syn. rhododendri* and three species of *Seimatosporium*, were selected according to the LSU phylogeny, and their ITS plus *β-tubulin* sequences were subjected to phylogenetic analysis using *Discosia artocreas* as the outgroup. The final dataset included 846 informative positions (522 bp of ITS and 335 bp of *β-tubulin*). The branch length of the NJ tree based on the analyses of ITS and *β-tubulin* was 0.227 (Figure S2B), and the highest log-likelihood of the phylogenetic tree generated using the ML method was −2131.68 (Figure 2). Using the MP method, a single most parsimonious tree was obtained with a tree length of 194 steps, and the consistency, retention, and composite indices were 0.832, 0.848, and 0.705, respectively, for parsimony-informative sites. The MP tree is shown in Figure S2A.

All phylogenetic trees clustered the Sporocadaceae into two clades (Figure 2). One clade comprised *Syn. rhododendri* and *Seim. foliicola* with maximum bootstrap support, with 94%–99% bootstrap support for the subclade comprising only *Syn. rhododendri*. The other highly supported clade comprised *Seim. botan* Sat. Hatak. & Y. Harada and *Seim. discosioides* (Ellis & Everh) Shoemaker.

### 3.3. Development of Conidiomata and Conidial Structures

**Figure 3:**
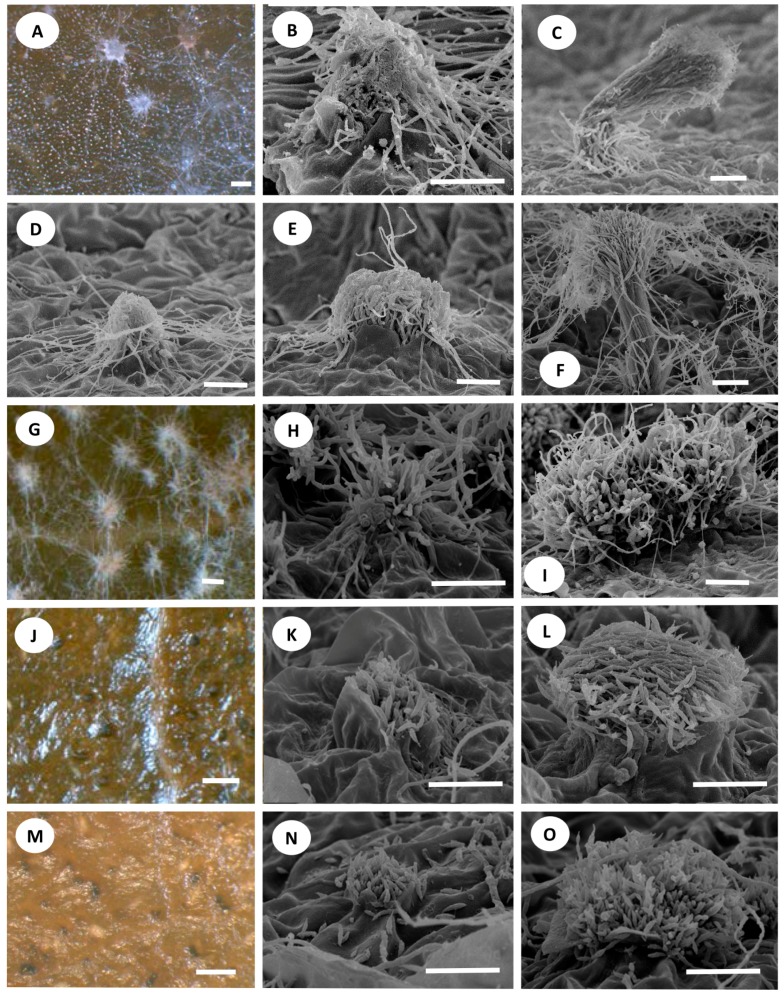
Ontogeny of conidiomata: (**A**–**C**) *Synnemapestaloides rhododendri* MAFF 239201; (**D**–**F**) *Syn. rhododendri* TAMA 492; (**G**–**I**) *Seimatosporium foliicola* NBRC 32676; (**J**–**L**) *Seim. botan* NBRC 104200; and (**M**–**O**) *Seim. discosioides* NBRC 104201. (**A**,**G**,**J**,**M**) Bars = 100 μm; (**B**–**F**,**H**,**I**,**K**,**L**,**N**,**O**) Bars = 50 μm.

The hyphal fascicles of the *Syn. rhododendri* strains burst through the epidermal cells of the plant leaf (MAFF 239201, Figure 3A,B; TAMA 492, Figure 3D,E). These hyphae elongated into synnematous conidiophores and produced conidia (MAFF 239201, Figure 3C; TAMA 492, Figure 3F). The ontogeny of *Seim. foliicola* (Figure 3G–I) was similar to that of *Syn. rhododendri*, except that its conidiophores were shorter (Figure 3I). Thus, the primordia of the conidiogenous cell of *Seim. foliicola* were not produced under the epidermal layers of the leaf (Figure 3G,H). In contrast, the conidia of *Seim. botan* (Figure 3J) and *S. discoides* (Figure 3M) did not appear until leaf surfaces cracked, and conidia were visible through breaks in the leaf surface (Figure 3K,L,N,O). This ontogeny is similar to those of *Pestalotiopsis guepinii* Desm., *P. neglecta* (Thüm.) Steyaert [24], and *Truncatella* species [25].

**Figure 4:**
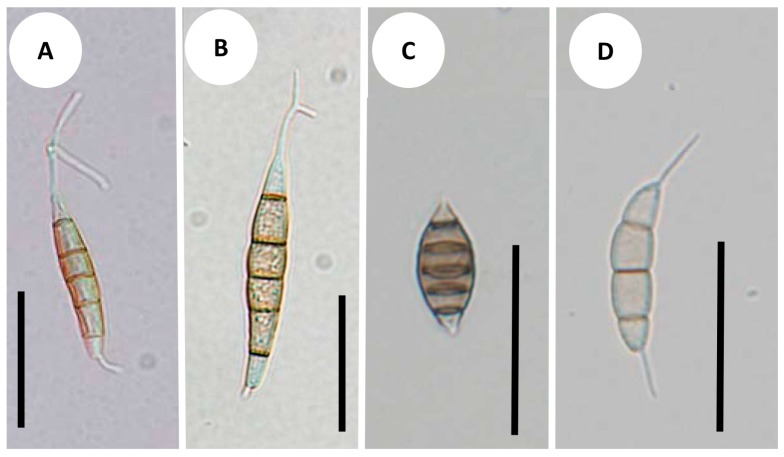
Conidial morphology: (**A**) *Synnemapestaloides rhododendri* MAFF 239201; (**B**) *Syn. rhododendri* TAMA 492; (**C**) *Seimatosporium foliicola* NBRC 32676; (**D**) *Seim. botan* NBRC104201. Bars = 25 μm.

*Synnemapestaloides rhododendri* and *Seim. foliicola* had conidia with six cells, including four pigmented median cells (Figure 4A–C). The conidia of *Seim. botan* (Figure 4D) and *Seim. discoides* had four cells, including two pigmented cells.

## 4. Discussion

Pestalotioid fungi share morphological features such as the masses of annelloconidia produced from conidiogenous cells inside or on their conidiomata, which are acervuli or pycnidia. Pestalotioid conidia are fusiform, straight, or slightly curved with several septa with or without simple or branched appendages at the apical or basal cells [26]. The first described pestalotioid fungus was *Pestalotia pezizoides* De Not., which grows on branches of *Vitis vinifera* L. [10], and its conidia with six cells, are fusiform, straight, or slightly curved. The basal appendage, which grows from truncated basal cells, is endogenous, unbranched, or dichotomously branched. Apical appendages are unbranched or dichotomously branched. Subsequently, fungi with different numbers of cells and appendages were classified as *Pestalotia*.

Since 1948, several studies have attempted to rearrange and separate more than seven related genera based on the number of the cells of the conidia and appendages [1,26,27,28] and variation in conidial ontogeny. Analysis of DNA sequences has also been used [3]. All morphologically related genera in this group were placed in the Amphisphaeriaceae within Amphishaeriales, but re-examination and reclassification at the genus and species levels are needed because these phylogenetic trees contained paraphyletic clades. Accordingly, Jaklitsch et al. [8] assigned this group to the Sporocadaceae within Amphishaeriales.

*Synnemapstalotiopsis rhododendri* and *P. pezizoides* produce similarly shaped conidia [9]. However, sporulation of *Synnemapestaloides* occurs on synnemata, and therefore the first strain that was isolated was classified as a new genus and designated as a hyphomycete [9] unlike other pestalotioid fungi which are coelomycetes. Hyde et al. [11] assigned *Synnemapestaloides* to the family Amphisphaeriaceae; however, this classification was not based on DNA sequence data. Since the suggestion of Hyde et al. [11], no published information has become available to confirm the inclusion of *Synnemapestaloides* in Amphisphaeriaceae. Here, our phylogenetic analysis assigned all strains of *Synnemapestaloides* and *Seimatosporium* to one clade, with relatively high bootstrap values (ML/MP/NJ: 94/82/92) for the phylogenetic tree obtained from the combined ITS-LSU matrix (Figure 1). The findings indicate that *Synnemapestaloides* belongs to the Sporocadaceae which had been separated from the Amphisphaeriaceae and mainly contain pestalotioid fungi.

In the present study, *Synnemapestaloides* formed a highly supported clade (ML/MP/NJ: 100/97/100) within the *Seimatosporium* clade, and the morphology of *Synnemapestaloides* was clearly distinguishable from that of *Seimatosporium* spp. The phylogenetic analyses of ITS and *β-tubulin* sequences (*D. artocreas* as the outgroup, Figure 2) allowed assignment of *Syn. rhododendri* and *Seim. foliicola* to a single clade with highest bootstrap support.

The species most closely related to *Syn. rhododendri*, was *Seim. foliicola*, and it was separated from other species of *Seimatosporium*, which supports the assignment of *Seim. foliicola* to its own genus separate from *Seimatosporium.* This species, which has conidia with five septa and single appendages at the apical and basal cells produced from conidiogenous cells on acervular, stromatic condiomata, is therefore grouped as the sarcostroma type [1]. The single major feature shared between *Seim. foliicola* and *Syn. rhododendri* was the cell number per conidium (Figure 4). Furthermore, we found that conidia from both fungi were produced from a structure resembling synnemata, although the lengths differed.

There are several developmental types of conidiomata [29]. Watanabe et al. [24] studied acervular development of the pestalotioid fungi *Pestalotiopsis neglecta* (Thüm.) Steyaert and *Truncatella* species. They found that the primordia of acervuli are produced as an aggregation of hyphae under the epidermis of a leaf, which develop into pycnidia-like structures that produce conidia from conidiogenous cells in the center of the cavity. The upper layer of this pycnidia-like structure is then disrupted by autolysis to form acervuli. Here we found that *Seim. botan* and *Seim. discoides*, which are grouped into *Seimatosporium*, produced conidia from conidiogenous cells under the epidermis, and conidia appeared when the leaf surface split (Figure 3K,N). This type of development is the same as that observed in studies of *Pestalotiopsis* spp. and *Truncatella* sp. [24,25] and differs from that of *Seim. foliicola.*

In contrast, the majority of conidiomata of *Syn. rhododendri* and *Seim. foliicola* grew upward and produced conidia at the tip of the conidiophore as synnemata on the leaf surface. The short synnemata (sporodochia) of *Seim. foliicola* can be easily confused with acervuli, time-lapse observations clearly discriminated between the conidioma types between the short synnemata (Figure 3H) and acervuli (Figure 3K, N). The synnemata of *Seim. foliicola* were shorter and wider compared with those of *Syn. rhododendri*, although the development of each was similar. For the clade shown in Figure 2 comprising *Syn. rhododendri* and *Seim. foliicola*, the synnema or sporodochia developing conidiophore may represent a monophyletic synapomorphic characteristic.

*Seimatosporium* is a large and diverse genus characterized by conidia comprising median pigmented cells with or without appendage(s). Classification within this genus has been discussed according to morphology as well as LSU and ITS sequences [1,6,26,27,30]. Molecular phylogenetic trees of *Seimatosporium* by Tanaka et al. [6] and Norphanphoun et al. [30] showed that *Seim. foliicola* is separated from other species. From this study, *Seim. foliicola* is closer to *Synnemapestaloides* than *Seimatosporium.*

In summary, we assign *Synnemapestaloides* to Sporocadaceae, and its most closely related genus is *Seimatosporium.* Molecular phylogenetic analyses and morphological analyses differentiate *Seim. foliicola* from other *Seimatosporium* species used in this study. Thus, *Seim. foliicola* should be transferred to *Synnemapestaloides*, and the formal taxonomic definition follows.

## Figures and Tables

**Table 1:** Strains of *Synnemapestaloides rhododendri* and closely related species with sequence source.

Species	Strain	GeneBank Accessions		
		ITS	LSU	*ß-tublin*
*Synnemapestaloides rhododendri*	MAFF 245156 ^1^	**LC047755 ^4^**	**LC047746**	**LC047763**
*Synnemapestaloides rhododendri*	MAFF 245157	**LC047756**	**LC047747**	**LC047764**
*Synnemapestaloides rhododendri*	MAFF 245158	**LC047754**	**LC047745**	**LC047762**
*Synnemapestaloides rhododendri*	MAFF 243052	**LC047757**	**LC047748**	**LC047765**
*Synnemapestaloides rhododendri*	MAFF 243053	**LC047758**	–	**LC047766**
*Synnemapestaloides rhododendri*	MAFF 243054	**LC047759**	–	**LC047767**
*Synnemapestaloides rhododendri*	TAMA 492 ^2^	**LC047760**	**LC047749**	**LC047768**
*Synnemapestaloides rhododendri*	MAFF 239201 *	**LC047753**	**LC047744**	**LC047761**
*Bartalinia laurina*	HKUCC 6537	AF405302	AF382369	–^5^
*Bartalinia pondoensis*	CMW 31067	GU291796	GU291796	–
*Bartalinia robillardoides*	CBS 122705	KJ710460	KJ710438	–
*Broomella vitalbae*	BV = CBS 140412	KT949895	KT949895	–
*Discosia* aff. *pleurochaeta*	MAFF24779	AB594781	AB593713	–
*Discosia artocreas*	NBRC 8975	AB594773	AB593705	–
*Discosia pini*	MAFF 410149	AB594776	AB593708	–
*Discosia pleurochaeta*	MAFF 242778	AB594777	AB593709	–
*Discosia tricellulare*	NBRC 32705 *	AB594796	AB593728	–
*Discosia yakushimense*	MAFF242774 *	AB594789	AB593721	–
*Discostroma tostum*	NBRC 32626	AB594795	AB593727	–
*Hymenopleella hippophaeicola*	LH = CBS 140410	KT949901	KT949901	–
*Immersidiscosia eucalypti*	MAFF 242781	AB594793	AB593725	–
*Lepteutypa fuckelii*	RS = CBS 131707	KT949902	KT949902	–
*Lepteutypa sambuci*	LEF = CBS 140409	KT949904	KT949904	–
*Monochaetia kansensis*	PSHI2004Endo1030	DQ534044	DQ534035	–
*Neoestalotiopsis protearum*	CBS 114178	JN712498	JN712564	–
*Neopestalotiopsis rosae*	CBS 101057	KM199359	KM116245	–
*Pestalotiopsis knightiae*	CBS 114138	KM199310	KM116227	–
*Pestalotiopsis malayana*	CBS 102220	KM199306	KM116238	–
*Phlogicylindrium uniforme*	CBS 131312	JQ044426	JQ044445	–
*Pseudopestalotiopsis cocos*	CBS 272.29	KM199378	KM116276	–
*Robillarda africana*	CBS 122.75	KR873253	KR873281	–
*Robillarda sessilis*	CBS 114312	KR873256	KR873284	–
*Seimatosporium biseptatum*	CPC 13584	JN871199	JN871208	–
*Seimatosporium foliicola*	NBRC 32676 ^3^	AB593734	AB594802	**LC047769**
*Seimatosporium hakeae*	NBRC 32678	AB594804	AB593736	–
*Seimatosporium obtusum*	CPC 12935 *	JN871206	JN871215	–
*Seimatosporium pistaciae*	CBS 138865	P004463	KP004491	–
*Seimatosporium botan*	NBRC 104200 *	AB594799	AB593731	**LC047770**
*Seimatosporium discosioides*	NBRC 104201	AB594800	AB593732	**LC047771**
*Seimatosporium eucalypti*	CBS 115131	JN871200	JN871209	–
*Seimatosporium hypericinum*	NBRC 32647	AB594805	AB593737	–
*Seimatosporium mariae*	NBRC 32681	AB594807	AB593740	–
*Seimatosporium parasiticum*	NBRC 32682	AB594805	AB593741	–
*Seiridium marginatum*	BLO = CBS 140403	KT949914	KT949914	–
*Seiridium phylicae*	CPC 19965	KC005787	KC005809	–
*Strickeria kochii*	C138	KT949917	KT949917	–
*Truncatella hartigii*	CBS 118148	DQ278913	DQ278928	–
*Zetiasplozna acaciae*	CBS 137994	KJ869149	KJ869206	–

*: EX-holotype culture, ^1^: MAFF: Genbank Project NARO, Japan; ^2^: TAMA: Culture collection of Tamagawa University; ^3^: NBRC: Biological Resource Center, NITE, Japan; ^4^: Accessions in bold were sequenced in this study; ^5^: This is not available; ITS: the internal transcribed spacer; LSU: the partial large subunit rRNA gene. Accession number in Bold were obtained in this study.

## Supplementary Materials

Supplementary materials can be accessed at: www.mdpi.com/2309-608X/2/4/28/s1.

## Appendix

### Taxonomy

#### ***Synnemapestaloides*** T. Handa & Y. Harada emend. Kyoko Watan., Nozawa, Kaz. Tanaka & Toy. Sato

Conidiomata synnematous or sporodochial, determinate, black, not changing color in 2% KOH or 85% lactic acid, arising from a basal stroma composed of textura angularis; hyphae of stipe parallel or those of sporodochia loose; conidial mass, black, globose to subglobose, subgelatinous. Conidiophores verticillately to sublaterally branched several times. Conidiogenous cells cylindrical to subcylindrical with annellations. Conidia fusiform with a truncate base, straight, septate, light olivaceous to pale brown; apical appendage centric; basal appendage excentric.

#### *Synnemapestaloides foliiola* (Berkeley) Kyoko Watan. Nozawa, Kaz. Tanaka & Toy. Sato comb. nov. [MB818747]

Basionym:

*Podisoma foliicola* Berk. in Smith The English Flora, Fungi 5-2 (2): 362 (1836).

Synonym:

≡*Hendersonia foliicola* (Berk.) Fuckel, Jahrbücher des Nassauischen Vereins für Naturkunde 23–24: 391 (1870).

≡*Cryptostictis foliicola* (Berk.) B. Sutton, Mycological Papers 88: 27 (1963).

≡*Seimatosporium foliicola* (Berk.) Shoemaker, Canadian Journal of Botany 42 (4): 416 (1964).

≡*Sarcostroma foliicola* (Berk.) M. Morelet, Bulletin de la Société des Sciences Naturelles et d'Archéologie de Toulon et du Var 37 (4): 233 (1985).

≡*Sarcostroma foliicola* (Berk.) Nag Raj, *Coelomycetous anamorphs* with appendage-bearing conidia: 787 (1993).
