# Supplementary material for: Phylogenetic Analysis of the Synnema-Producing Genus Synnemapestaloides
Source: J Fungi (Basel). 2016 Nov 7;2(4):28. doi: 10.3390/jof2040028 (PMC5715930; doi:10.3390/jof2040028)
Supplement: Supplementary file 1 [file jof-02-00028-s001.pdf]

Supplementary Materials: Phylogenetic Analysis of the Synnema-Producing Genus *Synnemapestaloides*

Kyoko Watanabe, Mao Sekiguchi, Toyozo Sato, Tom Hsiang, Shigeru Kaneko, Kazuaki Tanaka, Masaru Kanda, Naoko Fujita and Shunsuke Nozawa

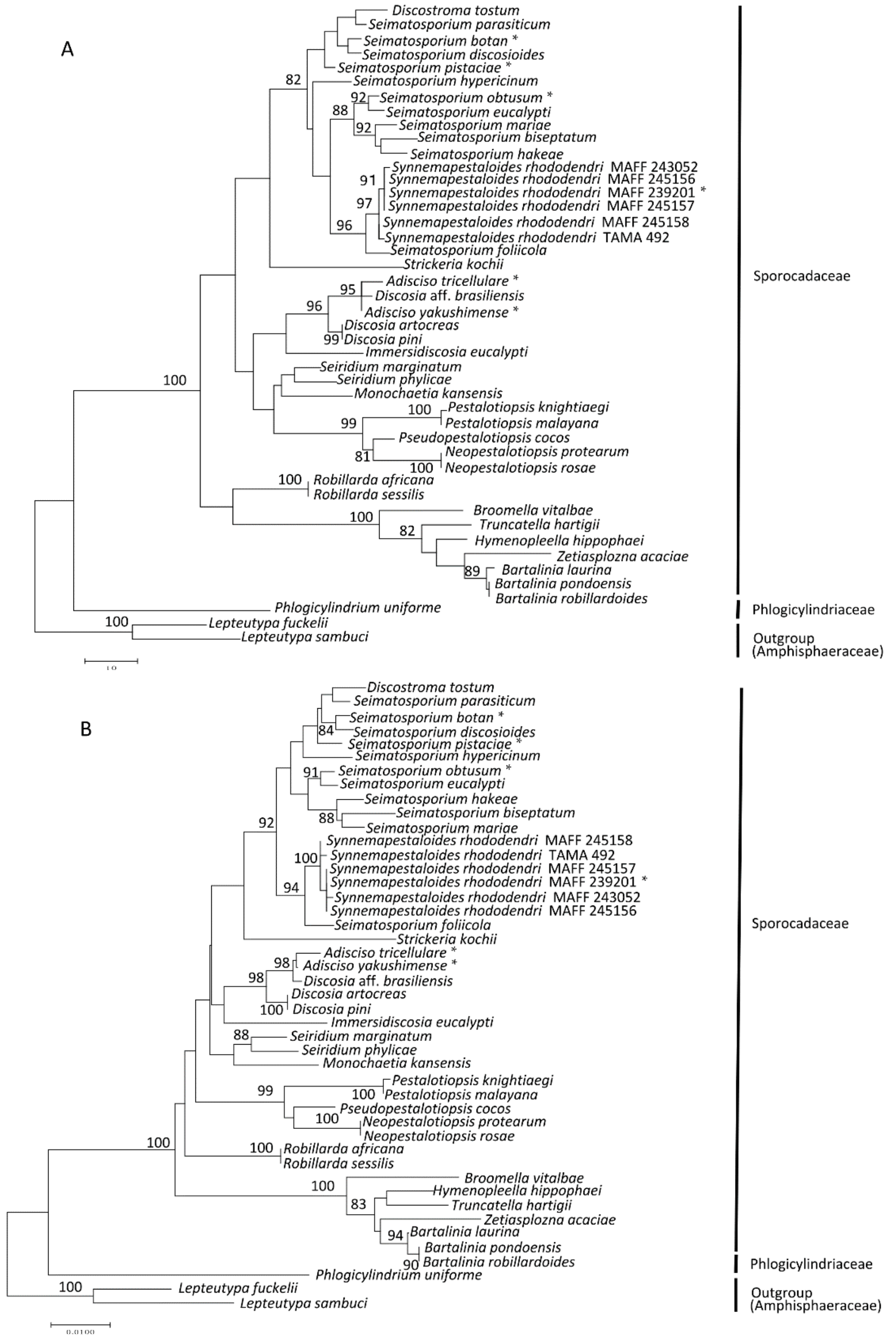

Figure S1. Phylogenetic trees based on analysis of ITS and LSU (D1–D2) sequences. Numbers on the branches indicate the bootstrap values (%) for each node, calculated from 1,000 replicates. (**A**), MP tree; (**B**), NJ tree. *: Ex-holotype cultures.

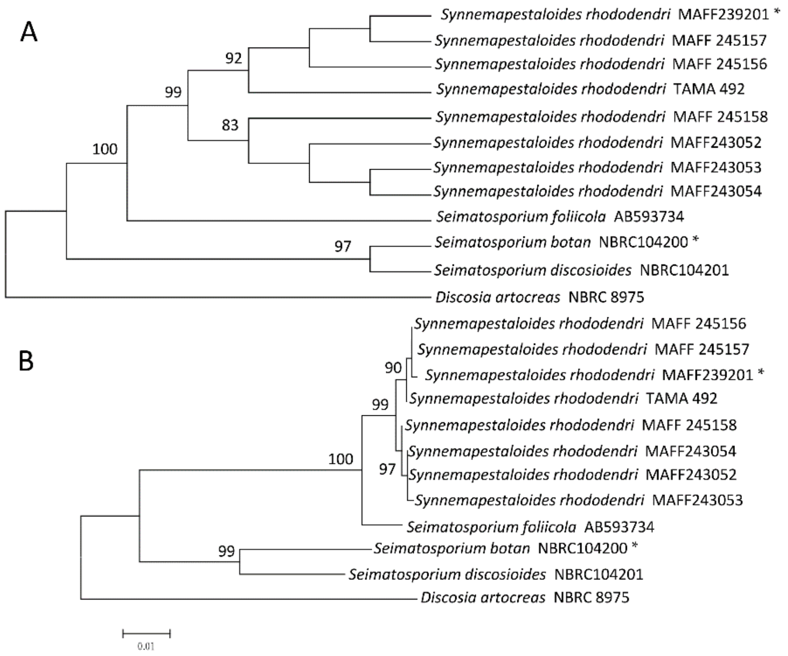

Figure S2. Phylogenetic trees based on ITS and *β-tubulin* sequences. The numbers on the branches indicate bootstrap values (%) for each node, calculated from 1,000 replicates. (**A**), MP tree; (**B**), NJ tree. *: Ex-holotype cultures.
